# Immune Responses of Dendritic Cells to Zoonotic DNA and RNA Viruses

**DOI:** 10.3390/vetsci12080692

**Published:** 2025-07-24

**Authors:** Xinyu Miao, Yixuan Han, Yinyan Yin, Yang Yang, Sujuan Chen, Xinan Jiao, Tao Qin, Daxin Peng

**Affiliations:** 1 College of Veterinary Medicine, Yangzhou University, Yangzhou 225009, China; miaoxy@yzu.edu.cn (X.M.); mx120231038@stu.yzu.edu.cn (Y.H.); chensj@yzu.edu.cn (S.C.); 2 Jiangsu Co-Innovation Center for the Prevention and Control of Important Animal Infectious Disease and Zoonoses, Yangzhou 225009, China; 3 Joint International Research Laboratory of Agriculture and Agri-Product Safety, The Ministry of Education of China, Yangzhou University, Yangzhou 225009, China; 4 Jiangsu Research Centre of Engineering and Technology for Prevention and Control of Poultry Disease, Yangzhou 225009, China; 5 College of Medicine, Yangzhou University, Yangzhou 225009, China; yyyin@yzu.edu.cn; 6 Guangling College, Yangzhou University, Yangzhou 225009, China; 7 International Research Laboratory of Prevention and Control of Important Animal Infectious Diseases and Zoonotic Diseases of Jiangsu Higher Education Institutions, Yangzhou University, Yangzhou 225009, China; yy@yzu.edu.cn; 8 Jiangsu Key Laboratory of Zoonosis, Yangzhou University, Yangzhou 225009, China; jiao@yzu.edu.cn

**Keywords:** dendritic cells, viral infection, immune response, zoonotic, DNA viruses, RNA viruses

## Abstract

Viral infections persistently challenge global health through immune evasion and zoonotic transmission. Dendritic cells (DCs) play a central role in antiviral immunity by detecting viral nucleic acids via conserved pattern recognition receptors, triggering interferon-driven innate responses and cross-presentation-mediated activation of cytotoxic CD8^+^ T cells. This study synthesizes DC-centric defense mechanisms against viral subversion, encompassing divergent nucleic acid sensing pathways for zoonotic DNA and RNA viruses, viral counterstrategies targeting DC maturation and interferon signaling, and functional specialization of DC subsets in immune coordination. Despite advances in DC-based vaccine platforms, clinical translation is hindered by cellular heterogeneity, immunosuppressive microenvironments, and limitations in antigen delivery. Future research should aim to enhance the efficiency of DC-mediated immunity, thereby establishing a robust scientific foundation for the development of next-generation vaccines and antiviral therapies. A more in-depth exploration of DC functions and regulatory mechanisms may unlock novel strategies for antiviral intervention, ultimately paving the way for improved prevention and treatment of viral infections.

## Simple Summary

Dendritic cells (DCs) serve as pivotal bridges connecting innate and adaptive immunity through the detection of viral nucleic acids mediated by pattern recognition receptors. DC subsets demonstrate specialized functions: conventional dendritic cells (cDCs) excel in cross-presentation, activating CD8^+^ T cells, while plasmacytoid dendritic cells (pDCs) dominate type I interferon production. Distinct immune recognition mechanisms emerge between DNA (TLR9/STING) and RNA viruses (TLR7/RIG-I), shaping differential cytokine profiles. DC-based vaccine strategies face dual challenges, including viral immune evasion mechanisms and cellular heterogeneity, in clinical translation.

## 1. Introduction

Viral infections persistently threaten global public health, as evidenced by recent outbreaks of highly pathogenic viruses that have caused substantial morbidity, economic disruption, and strain on the healthcare system worldwide [1]. The accelerating emergence of such pathogens is driven by anthropogenic factors, including climate change, ecological fragmentation, and intensified human–animal interfaces, which facilitate zoonotic spillover events that challenge existing immune defenses [2]. A fundamental dichotomy exists between DNA and RNA viruses: DNA viruses typically replicate in host nuclei, leveraging cellular transcriptional machinery while exhibiting lower mutation rates coupled with sophisticated epigenetic immune evasion tactics [3]. Conversely, RNA viruses replicate cytoplasmically with high error rates, enabling rapid antigenic drift and host adaptation [4]. Influenza viruses persistently illustrate the zoonotic emergence threat, evidenced by recurrent human spillovers of avian H5N1 and H7N9 strains since 1997 and pandemic origins of 1918 and 1957 outbreaks via avian–swine reassortment [5]. This review emphasizes influenza virology for its seminal contributions to understanding dendritic cell–virus coevolution across antigenically divergent strains.

Hosts counter these threats through layered immunity. Innate responses provide the first line of defense through pattern recognition receptors (PRRs) that detect pathogen-associated molecular patterns (PAMPs), such as viral nucleic acids, triggering interferon (IFN) responses that establish antiviral states in neighboring cells [6]. This primes adaptive immunity, where CD8^+^ cytotoxic T lymphocytes (CTLs) eliminate infected cells and B cells produce neutralizing antibodies [7]. Dendritic cells (DCs) serve as pivotal regulators, bridging these responses. Equipped with subset-specific PRRs, they capture viral antigens at infection sites, undergo maturation marked by the upregulation of major histocompatibility complex (MHC) and co-stimulatory molecules, and migrate to lymphoid organs to prime naïve T cells [8,9,10]. DC subsets exhibit functional specialization: conventional DCs (cDCs) demonstrate superior cross-presentation capacity to CD8^+^ T cells, while plasmacytoid DCs (pDCs) rapidly produce high-magnitude type I IFN upon viral encounter [11,12]. Viruses actively subvert DC functions—poxviral proteins inhibit PKR phosphorylation and influenza NS1 blocks RIG-I signaling—disrupting immune coordination to enable viral persistence [13,14].

This study systematically analyzes DC-mediated immune mechanisms against clinically significant viruses. Integrating advances in DC biology and viral immunoevasion, we establish a conceptual framework for developing DC-targeted vaccines and immunotherapies against emerging viral threats.

## 2. Immunological Basis of DCs

Generally, DCs are categorized into three main classes: cDCs, pDCs, and monocyte-derived DCs (Mo-DCs). As shown in Table 1, murine cDC1s are typically characterized as Lin-MHC-II^+^CD11c^+^CD8^+^ (resident cDC1s) or CD103^+^ (migratory cDC1s) [15,16]. Human cDC1s are characterized as Lin-CD64^−^HLA-DR^+^CD141^+^ cells [15]. The most notable function of cDC1s stems from their superior ability to cross-present cell-associated antigens, which primes CD8^+^ T cells, making cDC1s pivotal in antiviral and antitumor immune responses [17,18]. Murine cDC2s are characterized as Lin-MHC-II^+^CD11c^+^CD11b^+^SIRPα^+^ [19]. cDC2s in intestinal tissue also express CD103 [19,20]. Human cDC2s are identified as Lin-HLA-DR^+^CD1c^+^SIRPα^+^DCs [19,20]. cDC2s express various PRRs and can promote a wide range of immune responses, particularly CD4^+^ T-cell responses [21,22]. Moreover, one subset of cDC2s, known as inflammatory cDC2, has been identified in both mice and humans, characterized by distinct markers and transcriptional profiles that depend on the state of inflammation [16]. In human samples, this population, referred to as inflammatory cDC2 or DC3, is characterized by the expression of CD1c^+^CD5^-^CD14^+^ and variable CD163^+^ [23]. In mice, this population is characterized by the expression of CD64/MAR-1/Ly6C/CD14 [23]. Mo-DCs are characterized as MHC-II^+^ CD11c^+^ CD11b^+^CD64^int^Ly6C^int^CCR2^+^CD209^+^ in mice and HLA-DR^+^CD11c^+^CD14^int^CD206^+^ CD1c^+^ in humans [16]. pDC markers have been identified in both mice and humans. pDCs can regulate both innate and adaptive immune responses through IFN-I [24]. Mo-DCs can reactivate primed CD4^+^ T cells during states of inflammation [24]. pDCs orchestrate innate-to-adaptive immune bridging, primarily through rapid IFN-I production, establishing systemic antiviral states and modulating T/B-cell responses [24]. In contrast, cDCs specialize in antigen processing and presentation: cDC1s excel in cross-presenting exogenous antigens to CD8^+^ T cells via MHC-I, while cDC2s drive CD4^+^ T-cell activation through MHC-II-restricted presentation [25,26]. The presentation of endogenous cytosolic antigen on MHC II relies on autophagy [27]. Human DCs express high levels of MHC II and do not exhibit the key markers associated with T cells, B cells, natural killer (NK) cells, granulocytes, and monocytes. In human blood, DC subtypes include CD11c^+^ cDCs, which can be further categorized into CD141^+^ or CD1c^+^ cells, as well as pDCs, characterized by CD123^+^ cells [28]. DCs are a rare cell population in human tissues, and much of our understanding of their biology has come from studies in model organisms [29]. However, cross-species comparisons have highlighted significant differences between murine DC subsets and their human counterparts, including a lack of shared expression of subset-defining markers. This study presents a systematic evaluation of tissue-resident, ex vivo, and in vitro models of human DC biology by creating a comprehensive reference atlas. This atlas integrates data from multiple laboratories, diverse derivation methods, and various measurement platforms [30]. In murine DCs, cDCs can be divided into two subsets characterized by CD8α and CD103 or CD11b expression. Splenic CD8α^+^ cDCs are among the most efficient DC subsets in cross-presenting exogenous antigens via MHC-I to CD8^+^ T cells within lymphoid tissues, while CD103^+^ cDCs perform this critical function in non-lymphoid tissues such as mucosal barriers [31,32]. CD8α^+^ cDCs also present glycolipid antigens in the context of CD1d and can activate and polarize invariant natural killer T (iNKT) cells to produce Th1 or Th2 cytokines [33]. CD11b^+^ cDCs can be further classified based on additional surface markers, such as CD4, and endothelial cell-selective adhesion molecules (ESAMs) [34]. Compared to CD8α^+^ cDCs, CD11b^+^ cDCs are superior in inducing CD4^+^ T cell immunity, potentially due to their prominent expression of MHC II presentation machinery, which is attributed to their expression of the transcription factor IFN regulatory factor (IRF) 4 and cytokines such as IL-6 and IL-23 [34,35,36]. However, pDCs are best characterized by their unique ability to rapidly produce large amounts of type I interferons (IFN-I) in response to viral infections through the constitutive expression of IRF7 [37,38]. IRF3 acts as the master transcription factor for IFN-I production. Upon viral detection, TBK1 phosphorylates IRF3 at Ser386, triggering its dimerization and subsequent nuclear translocation. This induces transcription of IFNB and ISGs, establishing an antiviral state in infected and neighboring cells [39]. In human blood, except for pDCs, myeloid dendritic cells (mDCs) can be subdivided into three subsets using the surface markers CD16, CD1c, and BDCA-3. CD16^-^mDCs exhibit stronger pro-inflammatory activity, particularly in terms of TNF-α expression, which is 10- to 100-fold higher than that of CD1c^-^mDCs [40].

Liu et al. reported a novel murine DC lineage termed DC3, derived from Ly6C^+^ monocyte-dendritic cell progenitors (MDPs). These DC3s exhibit distinct ontogeny, phenotype, and function compared to classical cDC1/cDC2 subsets and monocytes, demonstrating a unique Th17-polarizing capacity in mice that may contribute to the development of inflammatory diseases. However, DC3s remain a murine-specific classification with no direct human equivalent established. Putative human counterparts are actively debated due to overlapping markers with monocytes and inconsistent functional data across tissues [41,42]. Myeloid lineage cells present in human peripheral blood include DCs and monocytes. Monocytes are broadly divided into two subsets: CD14^+^CD16^-^ (classical) and CD14dim CD16^+^ (nonclassical). A population of myeloid-derived cells that exhibit DC characteristics, namely HLA-DR^+^, and lack lineage markers, including CD14, but express CD16 is generally clustered with CD14^dim^ CD16^+^ monocytes [43]. Unlike their CD16^-^ counterparts, CD16^+^ monocytes rapidly differentiate into DCs upon reverse transendothelial migration and acquire an increased expression of co-stimulatory molecules when cultured in the presence of GM-CSF and IL-4 in vitro [44]. DCs derived from intermediate/nonclassical CD16^+^ monocytes, compared with classical CD16^-^ monocytes, exhibit a superior immune response during viral infections, such as HIV [45].

**Figure 1:**
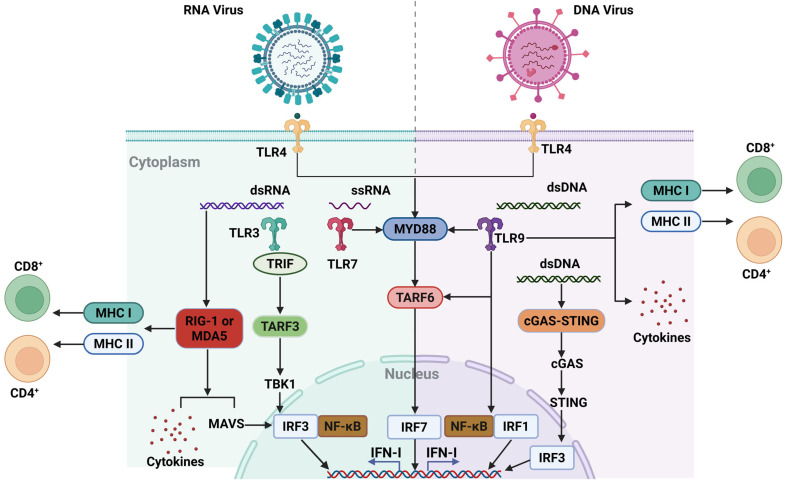
DC-centric immune recognition and mechanisms against zoonotic DNA and RNA viruses. The envelope proteins of DNA and RNA viruses activate TLR4, leading to the direct recruitment of MYD88. MYD88 recruits and activates TRAF6 through IRAKs. IRF7 is activated directly or indirectly by TARF6 in pDCs to induce IFN-I. After DNA viruses infect cells, their genomic dsDNA is degraded or released in lysosomes and endosomes. When TLR9 detects dsDNA, it changes shape and uses its intracellular TIR domain to recruit MYD88. The TLR9-MYD88 pathway can also induce IFN-I expression by activating IRF1 through TRAF6 and IKKα. TLR9 recognition also mediates the activation of NF-κB, leading to the upregulation of MHC I and MHC II. After RNA viruses infect cells, TLR3 and TLR7 recognize dsRNA and ssRNA, respectively, leading to the activation of IRF3 and IRF7. Upon recognizing dsRNA, TLR3 recruits TRIF, which triggers a kinase cascade involving TBK1, leading to the phosphorylation of IRF3. RIG-I and MDA5 recognize short and long dsRNA, respectively. Next, MAVS aggregation induces IRF3 phosphorylation, leading to the production of IFN-I. The activation of RIG-I and MDA5 increases the expression of MHC I and MHC II in DCs.

DCs recognize viral PAMPs and damage-associated molecular patterns (DAMPs) through PRRs such as Toll-like receptors (TLRs), triggering maturation and migration to the lymph nodes, where they activate adaptive immune responses. TLRs are a family of innate immune receptors, the activation of which is essential for initiating both innate and adaptive immune responses. The expression of TLRs in antigen-presenting cells (APCs) connects pathogen recognition to the activation of innate immune effector mechanisms that limit pathogen replication, as well as to the initiation of adaptive immunity [46]. TLRs detect conserved microbial features shared across a wide range of pathogen classes, allowing a limited set of receptors to recognize the vast diversity of microorganisms that the host may encounter. In DCs, three TLRs can be activated by nucleic acid ligands, referred to as NA-sensing TLRs: TLR3 recognizes double-stranded RNA (dsRNA); TLR7 recognizes specific fragments of single-stranded RNA with distinct sequence preferences; and TLR9 recognizes single-stranded DNA containing unmethylated CpG motifs (Figure 1). In DNA virus infections, DCs recognize viral DNA and glycoproteins, activating T cells, while the IFN-I produced by pDCs limits viral replication [47]. During RNA virus infections, DCs detect viral RNA through TLR 7 and RIG-I, activating the inflammasome and producing inflammatory cytokines and IFN-I to control viral spread. Viruses evade the immune system by interfering with DC functions, highlighting the importance of understanding the role of DCs in antiviral immunity, which provides a foundation for the development of vaccine and therapeutic strategies [48].

Upon viral recognition, DCs engulf viral particles through endocytosis with the assistance of the endoplasmic reticulum and Golgi apparatus, processing viral antigens into small peptides. These peptides then bind to MHC molecules. While antigens from DNA viruses are often presented to CD8^+^ T cells via MHC I molecules, and RNA virus antigens frequently utilize MHC II presentation to CD4^+^ T cells [49], both viral types can be presented through either MHC pathway depending on antigen source and cell type. When DNA and RNA viruses infect cells to replicate, their newly synthesized proteins produced in the cytoplasm are presented through MHC I. APCs internalize DNA and RNA virus particles and free viral proteins, subsequently presenting them via MHC II. Cross-presentation is a key mechanism that links viral antigens to MHC I, which enables APCs to initiate CD8^+^ T-cell responses against both DNA and RNA viruses, even if APCs are not infected [50,51,52]. APCs, especially DCs, are the bridge between the MHC I and MHC II pathways, which can activate both CD8^+^ and CD4^+^ T cells, initiating a complete antiviral immune response [51]. The presentation of MHC I molecules involves the degradation of viral antigens in the cytoplasm, entry into the endoplasmic reticulum via the antigen processing pathway, and binding to MHC I molecules before returning to the cell surface [53]. MHC II molecules, on the other hand, bind antigen peptides in the endoplasmic reticulum, form MHC II–antigen complexes, and are transported to the cell surface via the Golgi apparatus. When the MHC–antigen complexes on the surface of DCs bind to T-cell receptors (TCRs), DCs provide a second signal through co-stimulatory molecules (such as CD80, CD86, and CD40), thereby activating T cells [54]. This activation leads to the differentiation of CD8^+^ T cells into cytotoxic T cells that can recognize and kill virus-infected cells. In contrast, CD4^+^ T cells differentiate into helper T cells (Th cells), including Th1, Th2, Th17, follicular Th cells, and regulatory T cells, which further enhance the immune response by promoting antibody production by B cells and activating other immune cells [24]. Moreover, CD40, a member of the TNF receptor family, is constitutively expressed by mature DCs and other APCs and is upregulated during inflammation. It interacts with CD40L (CD145) on the surface of activated T cells to allow for T-cell priming and differentiation [24]. DCs also regulate the immune environment and enhance the antiviral immune response by secreting cytokines such as IFNs and interleukins (ILs) [55]. In particular, IFN-I plays a central role in antiviral immunity by promoting an antiviral state in cells and activating NK cells and other immune cells [56]. The entry of viruses into DCs utilizes various endocytic pathways, including three main mechanisms: clathrin-mediated endocytosis (CME); clathrin-independent endocytosis (CIE), including macropinocytosis; and phagocytosis. While phagocytosis is typically employed for larger particles, viruses smaller than 0.5–1 μm utilize the other two pathways. Macropinocytosis, an actin-dependent process, is constitutive in DCs and is exploited by many viruses for entry, including Vaccinia virus (VACV) and human adenovirus type 3. Many viruses utilize macropinocytosis to enter cells due to its constitutive nature in non-activated macrophages and DCs. Some viruses, like the Uukuniemi virus, utilize dendritic cell-specific intercellular adhesion molecule 3-grabbing non-integrin (DC-SIGN) for receptor-mediated endocytosis and subsequent trafficking to late endosomes before entering the cytosol [57].

As professional APCs, DCs effectively capture, process, and present viral antigens, activating and modulating adaptive immune responses in T and B lymphocytes [58]. In the early stages of viral infection, DCs recognize PAMPs and DAMPs of the virus through PRRs, triggering a series of immune reactions [59]. DCs are central to the production of cytokines and chemokines, which not only promote inflammation but also provide signals for the recruitment of other immune cells. For instance, DCs can secrete IFN-I, a key antiviral cytokine that enhances the antiviral state of cells and promotes the expression of antiviral genes through activation of the JAK-STAT signaling pathway [60]. DCs also express various chemokines, such as CCL2, CCL5, and CXCL10, which aid in recruiting monocytes and DC precursors from the circulatory system to the site of infection, further intensifying the local immune response [61]. It is generally acknowledged that the downstream signals of cGAS-STING, especially type I IFN, bridge innate immunity and adaptive immunity. Stimulated by cytosolic DNA, the active cyclic GMP-AMP synthase stimulator of the IFN gene pathway stimulates the expression of type I IFN in DCs, initiating innate immunity [62]. The cGAS-STING signaling pathway is the foundation of innate immunity against DNA viruses, where cytosolic viral DNA directly engages cGAS to trigger STING-dependent IFN-I responses [63]. Moreover, this pathway also significantly influences host defense against RNA viruses. Cells and mice deficient in cGAS or STING exhibit markedly increased susceptibility to a broad spectrum of RNA viruses, including flaviviruses and coronaviruses, accompanied by enhanced viral replication [64,65]. However, cGAS, as a dedicated DNA sensor, is unable to directly recognize RNA genomes. Many RNA viruses actively disrupt mitochondrial integrity, either through direct targeting of mitochondrial antiviral signaling protein (MAVS) or by inducing general cellular stress. This damage leads to the release of mitochondrial DNA (mtDNA) into the cytosol, where it serves as a potent ligand for cGAS, thereby activating STING and downstream IFN-I signaling [66,67]. For instance, Dengue virus NS2B protein promotes cGAS degradation, effectively weakening the defense of mtDNA-sensing mechanism [64]. Critically, the role of cGAS-STING in RNA virus infection is not uniformly protective. During infection with COVID-19, aberrant cGAS-STING activation driven by cytosolic DNA (potentially from host cell damage) contributes to the production of IFN-I and pro-inflammatory cytokines, aggravating the infection in the lungs [68]. This exemplifies the pathway’s paradoxical nature: it is indispensable for early viral containment under certain conditions, yet its aberrant activation can drive detrimental immunopathology in distinct contexts. Future research should precisely delineate the molecular mechanisms and kinetics of mtDNA and other DNA release triggered by specific RNA viruses.

The role of DCs in forming immune memory is also significant. Through effective antigen presentation, DCs activate specific T- and B-cell clones that can respond rapidly upon reencountering the same antigen, forming immune memory [69]. DCs facilitate the activation of CTLs through cross-presentation, a mechanism by which exogenous antigens are presented to CD8^+^ T cells, which is crucial for the clearance of virus-infected cells [70]. During the maturation of DCs, their phenotype and function undergo significant changes, including enhanced expression of antigen-presenting molecules such as MHC II and co-stimulatory molecules, thereby activating T cells more effectively [71]. Mature DCs can migrate to lymphoid organs, further activating T cells and promoting the unfolding of adaptive immune responses [72]. DCs act as a bridge between the innate and adaptive immune systems, making them an indispensable component of the immune response against viruses [73].

**Table 1:** Phenotype and function of DC subsets in humans and mice.

DC Subset	Phenotype in Humans	Phenotype in Mice	Function	Reference
cDC1	Lin-CD64^-^HLA-DR^+^CD141^+^	Lin-MHC II^+^CD11c^+^CD8^+^/CD103^+^	Cross-present soluble or cell-associated antigen; activate CD8^+^, Th1, and NK cells.	[15,17,18,22]
cDC2	Lin-HLA-DR^+^CD1c^+^SIRPα^+^	Lin-MHC-II^+^CD11c^+^CD11b^+^SIRPα^+^/CD103	Express various PRRs; activate CD4^+^ T cells.	[19,20,21,74]
Inflammatory cDC2	CD1c^+^CD5^−^CD14^+/-^CD163^+^	CD64/MAR-1/Ly6C/CD14	CD4^+^ T-cell priming; antigen cross-presentation; IFN-I stimulation.	[23,75,76]
Mo-DCs	HLA-DR^+^CD11c^+^CD14^int^CD206^+^CD1c^+^	MHC-II^+^CD11c^+^CD11b^+^CD64^int^Ly6C^int^CCR2^+^CD209^+^	Produce cytokines to shape differentiation of CD4^+^ T cell subsets.	[16,77,78]
pDCs	HLA-DR^+^CD11c^-^CD4^+^BDCA2^+^BDCA4^+^CD123 ^+^	MHC-II^int^CD11c^int^B220^+^Ly6C^+^BST2^+^SiglecH^+^	Induce T-cell proliferation after stimulation; produce IFN-I; regulate NK cell, DC, and Mφ survival; expand CD4^+^ T cells and CD8^+^ T cells.	[24,79,80,81]

## 3. Application of DC Immune Responses for Prevention and Control of Zoonoses

As shown in Table 2, DCs activate and mature upon recognition of PAMPs through PRRs, effectively presenting antigens to T cells, which is a crucial step in vaccine design [82]. For RNA viruses, such as influenza viruses and coronaviruses, DCs can recognize viral RNA through PRRs and activate antiviral responses [83]. In vaccine design, electroporation can be used to directly introduce mRNA-encoding viral antigens to DCs, causing them to express viral proteins and activate T cells [84]. Utilizing the cross-presentation ability of DCs, CD8^+^ T cells can be effectively activated to combat viral infections [85]. For DNA viruses such as HPV and HBV, vaccine design can leverage the antigen-presenting function of DCs [86]. Innate sensing of viral DNA within DCs promotes their maturation and activates T cells, thereby generating an immune response against the virus [87]. Through genetic engineering, viral antigens can be fused with DC-specific surface molecules to enhance the efficiency of antigen presentation. DC-based vaccine strategies can be designed for individualized tumor- or virus-specific antigens. By analyzing a patient’s tumor or viral sequences, custom mRNA or DNA vaccines can be developed to improve the specificity and efficacy of the vaccine [88]. To enhance the immune-activating capability of DCs, researchers have developed various vaccine adjuvants and delivery systems. For instance, using TLR agonists as adjuvants can enhance the maturation and antigen-presenting capacity of DCs [89]. Delivery systems, such as nanoparticles or virus-like particles, can enhance the stability and immunogenicity of vaccine components. Numerous clinical trials have demonstrated the safety and feasibility of DC-based vaccines in humans. Although most studies are small-scale, they provide valuable data for the further development of DC vaccines [90,91,92]. Future research needs to validate the clinical efficacy of DC vaccines in larger populations and explore optimal vaccine design strategies.

In the context of zoonoses, the strategic application of DCs may offer new perspectives for disease treatment. By promoting the generation of tolerogenic DCs (tolDCs), we can induce immune tolerance and reduce autoimmune and allergic reactions, which is particularly important for controlling chronic inflammation caused by zoonotic diseases [93]. By leveraging the regulatory functions of pDCs, we can modulate inflammatory cytokines in the synovial fluid of osteoarthritis patients, alleviating symptoms [94]. The application of DCs in immunotherapy is not limited to direct antigen presentation; they can also serve as carriers for immunotherapy, enhancing immune suppression by binding to specific immune regulatory molecules. Combined treatment strategies, such as using DCs in conjunction with other immunomodulators, can improve therapeutic effects. For example, combination with TGF-β can promote the proliferation and differentiation of chondrocytes [95]. The development of DC-based vaccines offers new possibilities for enhancing the immune response to specific zoonotic diseases [96]. In gene therapy, introducing immunomodulatory genes to DCs can make them more effective in treatment [97]. In combination with stem cell therapy, the co-administration of DCs with mesenchymal stem cells (MSCs) not only regulates immune responses but also promotes tissue repair [98].

Based on the influence of DCs on immune response, Qin et al. demonstrated that chitosan-functionalized iron oxide nanozyme (CS-IONzyme) promotes H1N1 whole inactivated virus (WIV) penetration across nasal mucosal barriers by recruiting submucosal DCs and inducing transepithelial dendrite (TED) formation through a TLR2/4^-^dependent pathway, thereby facilitating antigen uptake and immune activation [99]. DCs actively recruit to the submucosal layer through the secretion of CCL20, forming transepithelial dendritic networks. These dendritic projections penetrate epithelial barriers to directly capture viral particles, thereby enabling the acquisition of pathogens across membranes. During this process, CS-IONzyme potentiates DC function by activating ROS-dependent maturation pathways, upregulating co-stimulatory molecules (MHC II, CD40, CD80, CD86) to enhance antigen-presenting capacity while simultaneously increasing CD69 expression to sustain dendritic cell penetration activity [100]. This maturation process also involves the secretion of various cytokines, a reduction in endocytic capacity, and an enhanced ability to promote the proliferation of allogeneic CD4^+^ T cells. The ROS scavenger NAC can completely inhibit CS-IONzyme-induced DC maturation [99]. When a CS-IONzyme-based mucosal vaccine is applied, DCs can induce a strong adaptive immune response. This includes significantly increased titers of H1N1 WIV-specific mucosal IgA antibodies in nasal washes, tracheal washes, and lung washes of mice, enhanced expression of CD69 and proliferation index in spleen lymphocytes, and increased titers of HI antibodies and H1N1 WIV-specific IgG, IgG1, and IgG2a/c antibodies in serum, thereby inducing a Th2-type antibody response. As a mucosal immune adjuvant, CpG DNA enhances the transepithelial delivery efficiency of the WIV through unique mechanisms. Studies demonstrate that CpG DNA induces intestinal epithelial cells (IECs) to secrete the chemokine CCL20, which recruits lamina propria-resident CD103^+^ and CD103^-^ DCs to migrate toward the intestinal epithelium and form TEDs [101]. These TEDs directly capture luminal viral particles. Notably, the CD103^+^ DC subset exhibits a rapid migratory capacity, transporting antigens to mesenteric lymph nodes within 2 h to initiate antigen presentation, whereas CD103^−^ DCs primarily participate in local antigen uptake. This process does not rely on epithelial transcytosis or barrier disruption but instead enhances mucosal secretory IgA (sIgA) and systemic IgG responses (particularly Th1-biased IgG2a/c) by upregulating DC maturation markers (CD40, CD80, CD86, MHC II) and promoting the secretion of pro-inflammatory cytokines (IL-12, IL-23) [101]. In respiratory mucosa, CpG DNA similarly activates nasal epithelial barrier function via a CCL20-dependent pathway: inducing CD103^+^ DCs to penetrate tight junctions and form TEDs, which directly capture viruses through the SIGN-R1 PRRs, promoting DC maturation and migration to cervical lymph nodes for adaptive immune activation. Unlike cholera toxin (CT), which disrupts epithelial barrier integrity, CpG DNA enhances antigen presentation efficiency while preserving mucosal integrity [102]. Although vaccine strategies targeting DCs exhibit significant effects on animal models, suggesting promising applications in vaccine development, significant challenges limit the clinical translation of DC-based therapies. These limitations include cellular heterogeneity, immunosuppressive microenvironments, and disparities between animal models and human trials. As shown in Table 1, distinct subsets of human DCs present different phenotypes and functions compared with mouse DCs, which cannot replicate human DC biology in animal models. For instance, pDCs in humans produce 10–100-fold more IFN-α than those in mice upon TLR7 activation, complicating dose extrapolation [103]. Murine models show robust memory T-cell responses following DC vaccination, but humans exhibit shorter-lived immunity due to homeostatic constraints [104]. Viral infection and tumors create microenvironments that destroy DC function, impairing antigen presentation. Viruses like MPXV (via M2 protein) and influenza (via NS1) inhibit DC maturation, MHC expression, and cytokine production [105,106]. Lactic acid and hypoxia induced by tumors inhibit DC glycolysis and antigen processing, limiting vaccine efficacy [107]. Although DC-based vaccines show modest success in cancer (e.g., Sipuleucel-T for prostate cancer), clinical trial failures often occur in viral infection. Human trials of influenza vaccines targeting DCs generated antibody titers but often lacked cross-protection against drifted strains [108]. In addition, DC vaccines for HIV produced CD8^+^ T-cell responses but failed to produce lasting immunity due to viral latency and immune escape [109]. Based on a summary of the applications of DC immune responses (Table 3), future strategies for DC vaccines should focus on overcoming the differences between organisms and using combined vaccines to improve the efficiency of adjuvants to promote the application of DC vaccines.

**Table 2:** PAMPs and PRRs during viral infection in DCs.

Type of Virus	Virus	PAMPs	PRRs	Reference
DNA viruses	CPXV	HA glycoprotein, A52, B15, K7	TLR3	[110,111,112]
	MPXV	A47, F14L, F3	TLR4, TLR9, PKR, MDA-5, RIG-I	[113,114,115,116]
RNA viruses	SARS-CoV	S protein	DC-SIGN	[117,118]
	MERS-CoV	S protein	DPP4	[119,120]
	IAV	HA, NA, NS1, NP, PA-X	TLR3, TLR4, TLR7, RLRs, DC-SIGN, SIGN-R1	[59,102,105,121,122,123,124,125]

**Table 3:** DC applications in vaccine development.

Application	Core Strategy	Key Mechanism	Advantage	Challenge
RNA virus vaccines	mRNA electroporation	Viral antigen expression → cross-presentation → CD8^+^ T-cell activation	Direct DC targeting; cellular immunity	In vivo delivery efficiency
DNA virus vaccines	DNA sensing activation	Viral DNA detection → DC maturation → T-cell response	Enhanced targeting via engineering	Antigen design complexity
Adjuvant systems	TLR agonists/nanoparticles	Boosts DC maturation; stabilizes antigens	Overcomes immune tolerance	Weaker response in humans
Mucosal immunization	DC recruiters (CpG/CS-IONzyme)	TED formation → transepithelial antigen capture → mucosal IgA	Enhances barrier immunity; preserves integrity	Significant species differences
Immune regulation	tolDC induction	tolDCs suppress inflammation; pDCs modulate tissue damage	Controls chronic disease inflammation	Immunosuppressive microenvironments
Combo therapies	DCs ^+^ immunomodulators/MSCs	Synergistic effects (e.g., TGF-β ^+^ chondrocyte regeneration)	Integrated immune repair	Mechanism complexity
Future development	Multi-adjuvant vaccines	Overcoming species gaps; breaking immunosuppression; durable immune memory	Broad-spectrum prevention potential	Requires humanized models

## 4. Immune Recognition and Response to DNA Viruses

After DNA viruses enter host cells, DCs, as key innate immune cells, play a crucial role in recognizing and responding to viral invasion through PRRs (Figure 1). In dealing with DNA viruses, DCs present viral antigens to CD8 T cells through MHC I molecules, inducing the production of CTLs that can recognize and eliminate infected cells [126]. DCs present antigens to CD4 T cells via MHC II molecules, activating Th cells that further secrete cytokines such as IFN-γ and TNF-α, promoting the antiviral immune response [127]. The cytokines and chemokines secreted by DCs during antigen presentation play a crucial role in regulating the immune response. For example, the secretion of IL-12 promotes the differentiation of Th1 cells, thereby enhancing the cytotoxic immune response, while the production of IL-10 helps regulate the immune response and prevent excessive inflammatory damage [128]. DCs also attract CCR5^+^ CD8^+^ T cells to the site of infection by secreting chemokines such as CCL3, CCL4, and CCL5, enhancing the local immune response [129]. In the specific immune response against DNA viruses, the role of DCs is not limited to activating T cells; they also participate in regulating the formation of immune memory [104]. After DNA virus infection, DCs activate memory T cells through cross-presentation mechanisms, providing rapid and effective immune protection upon reencountering the same virus [130]. However, orthopoxviruses sabotage these defenses through F14 protein binding to phosphorylated Ser386 on IRF3 [131], suppressing IFN-β production and crippling DC maturation, while K7 protein induces mtDNA leakage, hyperactivating cGAS-STING to drive IL-6/TNF-α-dominated inflammation [132]. These coordinated strategies disrupt antigen processing, co-stimulation, and DC survival, ultimately impairing cross-presentation and T-cell priming to facilitate viral persistence in host tissues.

### 4.1. Cowpox Virus (CPXV)

Cowpox virus (CPXV) has a dsDNA genome and is classified within the poxviridae family, renowned for its crucial role in the development of the smallpox vaccine [133]. CPXV particles have a characteristic brick-like shape, and their lifecycle primarily takes place in the cytoplasm of host cells [134]. CPXV infects hosts through various routes, including inhalation, direct contact with the skin or mucous membranes, and entry through damaged skin [134]. After infection, the virus interacts with DCs, which play a central role in antigen presentation and immune activation [135]. The CPXV recognizes and enters DCs, utilizing its surface proteins, such as the hemagglutinin glycoprotein, to bind to receptors on DCs [111]. Although CPXV infection in DCs is nonproductive and fails to kill the cells, it profoundly suppresses both innate and acquired immune functions of DCs, including cDCs and pDCs [106]. The virus replicates within DCs, affecting the maturation and antigen-presenting capabilities of these cells. The initial response of DCs to CPXV infection involves the secretion of inflammatory cytokines such as TNF-α and IL-6, which facilitate the recruitment of other immune cells to the site of infection [136]. CPXV has also evolved multiple strategies to evade the host’s immune response, including inhibiting the migration of DCs and interfering with the antigen presentation process [137].

In the early stages of infection, CPXV can activate DCs, prompting them to mature and migrate to the lymph nodes [138]. CPXV has also evolved various strategies to interfere with DC functions, affecting their antigen-presenting abilities. Virally encoded immunomodulatory proteins, such as A52, B15, and K7, can target the NF-κB signaling pathway, thereby inhibiting the expression of pro-inflammatory cytokines and chemokines and evading immune control [112]. CPXV infection of DCs inhibits LPS-induced cytokine mRNA synthesis and nuclear translocation of NF-κB. A representative CPXV immune evasion protein, CrmA, is also detected in DCs 2 h post-infection [106]. CPXV blocks IFN signaling through encoded receptor homologs such as IL-1β, TNF-α, IFN-α, and IFN-β, thereby inhibiting the maturation and migration of DCs. Some mature DCs can maintain the expression of co-stimulatory molecules after infection and directly present CPXV antigens to activate T cells. After infection with CPXV, DCs also produce a series of cytokines and chemokines that play important roles in regulating the immune response. For example, CPXV infection can induce the production of TNF-α through its receptors TNFR1 and TNFR2 [106,139]. CPXV neutralizes TNF-α through its encoded homolog of TNF receptor 1 (vTNFR1), blocking its function in skin cells and thus interfering with the inflammatory response and the recruitment of immune cells. The infection of DCs with CPXV stimulates the host’s immune response, while the virus also employs multiple mechanisms to suppress and regulate this process in order to maintain its survival and spread within the host [140].

### 4.2. Monkeypox Virus (MPXV)

Classified as a zoonosis, monkeypox disease results from infection with monkeypox virus (MPXV), a dsDNA pathogen belonging to the Poxviridae family and Orthopoxvirus genus [141]. MPXV exhibits approximately 90% genetic homology with the variola virus, the causative agent of smallpox. Consequently, vaccines and therapeutics developed for smallpox confer significant protection against MPXV infection [142]. Infection with MPXV triggers an adaptive immune response that can effectively control disease progression. The primary routes of MPXV entry into the host organism are through the respiratory tract or breaches in the skin barrier [143]. APCs, including DCs and macrophages, represent susceptible targets for MPXV infection [144]. MPXV infection both activates and subsequently subverts cytosolic DNA sensing mechanisms. These sensors detect cytoplasmic dsDNA, triggering signaling cascades that involve NF-κB and IRF3/IRF7. This normally results in the secretion of pro-inflammatory cytokines, including IFN-I and IFN-III, to mount an antiviral defense [113]. TLR9 expressed on DCs plays a vital role in detecting MPXV and subsequently promoting the recruitment of NK cells to sites of infection [113]. Essential for conferring IFN resistance, MPXV expresses the F3 protein, an ortholog of the VACV E3 protein. MPXV infection induces aberrant transcriptional termination during intermediate and late phases, generating extended viral RNA transcripts with complementary regions that undergo intramolecular or intermolecular hybridization, ultimately leading to measurable intracellular accumulation of dsRNA duplexes [145]. The F3 protein functions by binding dsRNA and sequestering it from recognition by PRRs such as MDA-5, RIG-I, and PKR, consequently inhibiting their activation [114]. Specifically, MPXV F3 inhibits the dsRNA-dependent phosphorylation of PKR and its substrate, eIF2α, resulting in the suppression of protein translation and a reduction in IFN production [114,145]. In addition to the PKR pathway, a recent study demonstrated that MPXV protein p2 interacts with karyopherin α-2 (KPNA2) to promote its nuclear translocation while competitively inhibiting KPNA2-mediated IRF3 nuclear translocation and suppressing downstream IFN production [146]. MPXV expresses A47, a functional homolog of the VACV A52R protein known to inhibit TLR3 and TLR4 signaling. VACV additionally utilizes its A46 protein to specifically target TLR4 signaling. IFNs are pivotal for suppressing viral replication. To counteract this, MPXV secretes the B16 protein, acting as a soluble antagonist of the IFN-II signaling cascade. MPXV further subverts host immunity by targeting key antiviral cytokines, such as TNF-α, and other immunomodulators. MPXV encodes cytokine response-modifying protein B (CrmB), which acts as a decoy receptor for TNFα, effectively neutralizing its activity and disrupting immune activation [116,147].

Beyond innate immunity evasion, MPXV undermines adaptive T-cell responses (both CD8^+^ and CD4^+^) by interfering with T-cell receptor-mediated activation, potentially through mechanisms involving altered antigen presentation [148]. Human MPXV infection is characterized by decreased levels of TNF-α, IFN-γ, IL-2, and IL-12, concurrent with elevated concentrations of certain ILs and chemokines, including CCL2 and CCL5. Infection elicits the production of IgM and IgG antibodies. While IgG^+^ memory B cells persist in the long term, activated effector CD4^+^ and CD8^+^ T cells undergo rapid initial proliferation followed by a gradual decline [147]. The MPXV B22 protein hinders T-cell-mediated viral containment. Simultaneously, the MPXV M2 protein suppresses T-cell activation by binding B7 ligands (CD80/CD86), thereby blocking the essential CD28 co-stimulatory signal with greater potency than CTLA-4. Collectively, this dual inhibition markedly reduces the activation of CD4^+^ and CD8^+^ T cells, consequently disrupting germinal center development and B-cell maturation—critical steps in generating a potent adaptive immune response. Furthermore, the M2 protein potentiates PD-L1 signaling, thereby exacerbating T-cell exhaustion and contributing to broader immune dysfunction. Significantly, the Modified Vaccinia Ankara-Bavarian Nordic (MVA-BN) vaccine strain lacks a functional M2 protein [115,116]. This absence correlates with stronger CD4^+^ and CD8^+^ T-cell responses compared to those elicited by MPXV or wild-type VACV infection. Chen et al. investigated antigen-specific T-cell responses at the single-cell level following natural MPXV infection and vaccination. Their findings demonstrate that T-cell immunity predominantly targets viral early expressed proteins. Although tetramer-positive CD8^+^ T cells exhibit comparable differentiation and activation phenotypes in both cohorts, T cells from convalescent individuals display enhanced cytotoxicity, greater migratory capacity toward infection sites, and broader TCR clonal expansion [149]. These observations highlight the potential of genetic engineering approaches to remove inhibitory genes, such as M2, from MVA-based vectors, thereby enhancing their vaccine efficacy by fostering potent T-cell-mediated immunity against MPXV.

cGAS serves as a broad-spectrum cytoplasmic DNA sensor, detecting viral DNA from diverse sources (including DNA viruses and retroviruses), as well as microbial and tumor-derived DNA [150]. Activation of the cGAS-STING pathway subsequently triggers both the NF-κB signaling pathway and the IFN production pathway. Wild-type VACV disrupts the cGAS-STING-NF-κB axis through its F14 protein (encoded by the F14L gene). F14 mimics the transactivation domain of the NF-κB p65 subunit, selectively inhibiting the expression of NF-κB-dependent antiviral genes. The F14 protein exhibits high conservation across orthopoxviruses (OPVs). The MPXV F14 homolog shares 98.7% amino acid identity with VACV F14. Moreover, the transcriptional regulatory elements flanking the F14L open reading frames (ORFs) are also highly conserved among OPVs [115,151]. Therefore, it is highly probable that MPXV utilizes mechanisms analogous to those of VACV to inhibit the cGAS-STING-NF-κB signaling pathway. In summary, current research demonstrates that MPXV deploys a sophisticated array of immune subversion and evasion strategies. These mechanisms facilitate viral persistence and dissemination within the host, potentially contributing to heightened clinical disease severity.

## 5. Immune Recognition and Response to RNA Viruses

RNA viruses employ various strategies to enter host cells, with examples such as SARS-CoV-2 utilizing its spike protein (S protein) to bind the host cell surface receptor angiotensin-converting enzyme 2 (ACE2) [152]. Once inside the cell, the positive-sense, single-stranded RNA genome of the virus is directly translated by the host cell’s ribosomes, producing viral nonstructural proteins, such as RNA-dependent RNA polymerase (RdRp), and structural proteins. RdRp then catalyzes the synthesis of a complementary negative-strand RNA template from the genomic RNA, forming dsRNA replicative intermediates. Subsequently, these intermediates serve as templates for the synthesis of multiple new positive-sense genomic RNAs. Finally, the newly synthesized viral genomes, together with the structural proteins, assemble into infectious virus particles. [153].

DCs utilize the expression of multiple PRRs to recognize RNA viruses [154]. Particularly, PRRs in the endoplasmic reticulum and cytoplasm, such as RIG-I and MDA5, can directly recognize viral RNA [155] (Figure 1). These receptors are highly sensitive to the double-stranded structure or 5′-triphosphate group of viral RNA, triggering rapid immune responses [156]. The activation of these PRRs leads to the activation of downstream signaling molecules such as MAVS and TBK1, which then activate transcription factors like NF-κB and IRF3 [157]. This promotes the production of IFN-I and other inflammatory factors, thereby initiating the antiviral immune response. RNA virus recognition also leads to the maturation of DCs from an immature to a mature state, enhancing their antigen-presenting capabilities [158]. PRRs participate not only in the initial recognition and signaling of viruses but also in the regulation of immune responses [159]. For example, TLR3, TLR7, and TLR8, in addition to directly recognizing viral RNA, can influence the local and systemic immune environment by regulating cytokine production [160]. The activation of PRRs plays a crucial role in virus clearance. The activation of TLR7 can promote pDCs to produce large amounts of IFN-α, which is crucial for limiting the replication and spread of certain RNA viruses [161].

### 5.1. Severe Acute Respiratory Syndrome Coronavirus (SARS-CoV)

SARS-CoV is a single-stranded positive-sense RNA virus belonging to the Coronaviridae family, known for its high infectivity and pathogenicity [162]. SARS-CoV shares >96% nucleotide identity with certain bat SARS-like coronaviruses. Its genome is a single-stranded, positive-sense RNA molecule of 29.7–29.9 kilobases, organized into 14–15 ORFs. These ORFs encode 16 nonstructural proteins, 4 structural proteins, and 6–9 accessory proteins [163]. The virus infects not only humans but also a variety of mammals, demonstrating a broad host range. The infection pathway of SARS-CoV is primarily achieved through the binding of its surface S protein to the ACE2 receptor on the host cell surface. This binding triggers the fusion of the virus with the cell membrane, allowing the viral genetic material to enter the host cell. SARS-CoV-2 enters DCs and macrophages through DC-SIGN and furin rather than through ACE2 and TMPRSS2 [118]. Recent studies have demonstrated that SARS-CoV binds to DC-SIGN through its activated S protein, triggering an inflammatory response in DCs [117]. Moreover, a recent study showed that DC-SIGN myeloid cells possess a large array of C-type lectins (CLRs) capable of interacting with the SARS-CoV-2 S protein, which includes liver/lymph node-specific intercellular adhesion molecule-3-grabbing integrin (L-SIGN), liver sinusoidal endothelial cell lectin (LSECtin), asialoglycoprotein receptor 1 (ASGR1), and CLEC10A [164]. Interestingly, these pattern recognition receptors cannot lead to active viral infection or replication, which is different from ACE2. Why these receptors, unlike ACE2, do not support active infection warrants further investigation. Therefore, whether SARS-CoV-2 can directly infect DCs by binding to CLRs independently of ACE2 is still an open question. Neuropilin-1 has been recently identified as an unconventional receptor for SARS-CoV-2, as it binds to the S1 subunit of the S protein generated by furin [165]. Another study revealed a novel route of SARS-CoV-2 infection through the engagement of CD147 by the spike protein [166]. Notably, Neuropilin-1 and CD147 are well-expressed by DCs [167], which makes them potential receptors for SARS-CoV-2 to enter DCs. The expressions of CD147 and ACE2 are exclusively independent in single lung cell [166], suggesting that CD147 and ACE2 may be two complementary receptors in mediating virus infection. Therefore, future specific immunization strategies for COVID-19 prevention can target CD147 on DCs. SARS-CoV is also capable of inducing pyroptosis in DCs, a caspase-1-dependent form of programmed cell death that results in the formation of pores in the cell membrane, cell swelling, and osmotic lysis, thereby promoting virus release and exacerbating the inflammatory response [168]. Studies have shown that SARS-CoV-infected DCs may have abnormalities in activating inflammasomes and inducing cytokine release, which may be related to the virus’s mechanism of evading host immune surveillance [117]. The above effects on DCs during infection may impair antigen-presenting capacity, affecting T-cell activation and the initiation of the immune response. Although SARS-CoV can induce DCs to produce IFN-I, certain viral proteins may weaken the host’s antiviral response by inhibiting IFN-I signaling. Viral nonstructural proteins (e.g., nsp1, nsp3) and accessory proteins (e.g., ORF3b, ORF6) impair IFN-I synthesis or interfere with JAK-STAT signal transduction [169,170]. Notably, ORF6 binds karyopherin α2 (KPNA2) to inhibit STAT1 nuclear translocation, while the papain-like protease (PLpro) domain within nsp3 suppresses IRF3 and NF-κB pathway activation through its deubiquitinating/deISGylating activity [171,172]. The interaction between SARS-CoV and DCs involves not only direct infection and replication but also complex regulation of the host immune response.

Following SARS-CoV infection, DCs undergo a rapid process of activation and maturation, accompanied by the upregulation of antigen-presenting molecules such as MHC and co-stimulatory molecules [173]. Unlike conventional activation, SARS-CoV-infected DCs may exhibit abnormalities during maturation, such as altered cytokine production patterns and reduced capacity for T-cell activation. SARS-CoV can induce DCs to produce anti-inflammatory cytokines, including IL-10, while decreasing the production of pro-inflammatory cytokines, such as IL-12, which may lead to the suppression of the immune response and a decrease in viral clearance capacity [174]. The impact of SARS-CoV on the immune regulatory functions of DCs is also evident in its interference with co-stimulatory signals. Studies have shown that SARS-CoV-infected DCs may downregulate the expression of co-stimulatory molecules such as CD80 and CD86, thereby affecting T-cell activation and proliferation [175]. Collectively, these abnormalities represent a viral strategy to evade host defenses by disturbing DC immune response. The virus may also weaken the adaptive immune response by interfering with the migratory ability of DCs, thereby affecting their localization in lymph nodes and their interaction with T cells. Studies have shown that the immunological memory induced by SARS-CoV infection develops after natural infection and persists for at least 6 to 8 months [176,177]. While DCs theoretically can promote viral clearance, SARS-CoV infection may lead to impaired DC function, which reduces effective antigen-specific T-cell responses and thus affects the efficiency of viral clearance [178]. The immune regulatory imbalance caused by SARS-CoV infection may be related to the severity and progression of the disease.

### 5.2. Middle East Respiratory Syndrome Coronavirus (MERS-CoV)

MERS-CoV is a single-stranded, positive-sense RNA virus belonging to the Coronaviridae family, with a genome length of approximately 30 kilobases, capable of infecting a variety of mammalian hosts, including humans [179]. MERS-CoV infection typically initiates with the binding of the viral S protein to the dipeptidyl peptidase 4 (DPP4) receptor on the host cell’s surface, which is crucial for the virus to enter the host cell. MERS-CoV triggers membrane fusion through its interaction with DPP4, enabling the release of viral genetic material into the host cell and initiating its replication cycle [180]. Although both SARS-CoV and MERS-CoV infect monocyte macrophages, DCs, and activated T cells, only MERS-CoV is able to replicate in the infected immune cells, which consequently results in the aberrant induction of inflammatory cytokines in macrophages and DCs [120]. MERS-CoV can enter DCs using the DPP4 receptor on their surface, a process that not only promotes viral spread but also affects DC function and antigen-presenting capacity. After MERS-CoV infects DCs, it may interfere with their normal maturation process, leading to impaired antigen-presenting function and thus affecting T-cell activation and expansion [119]. The interaction between MERS-CoV and DCs also involves viral immune regulatory effects on DCs. Virus infection may induce DCs to produce specific cytokines and chemokines that play a role in regulating both local and systemic immune responses. MERS-CoV may also inhibit the host’s immune response by interfering with DC signaling pathways, thereby affecting the host’s capacity to produce IFNs and other anti-inflammatory cytokines. The infection strategy of MERS-CoV relies not only on its interaction with host cell receptors but also on the regulation of the host immune system, especially DCs [181].

Following MERS-CoV infection, the activation and maturation of DCs are affected, resulting in a reduction in their antigen-presenting capacity. MERS-CoV can infect human primary DCs and affect their survival and function by activating both extrinsic and intrinsic apoptotic pathways [120]. The S protein of MERS-CoV, through DPP4-mediated signaling, inhibits the expression of IFN-I, thereby weakening the host’s antiviral response. The impact of MERS-CoV on the immune regulatory functions of DCs is multifaceted. MERS-CoV alters the cytokine profile of DCs by suppressing the production of cytokines, particularly IFN-I [182]. This alteration leads to the suppression of T-cell activation, thereby affecting the initiation and maintenance of adaptive immune responses. DCs infected with MERS-CoV exhibit changes in co-stimulatory molecule expression, which may affect their ability to activate T cells. The overall impact of MERS-CoV infection on DC-mediated immune responses is complex. Although the virus strategically impairs DC function, the host’s immune system still attempts to clear the virus through other mechanisms. For example, specific T-cell responses have been observed in individuals infected with MERS-CoV, with these T cells being able to recognize multiple viral proteins [183]. Viral immune evasion mechanisms may lead to disease progression and exacerbation of clinical symptoms. By interfering with the maturation, antigen presentation, and immune regulatory functions of DCs, MERS-CoV compromises the host’s antiviral immune response, thereby creating conditions that facilitate the virus’s persistence and spread.

### 5.3. Influenza A Virus (IAV)

Influenza viruses, as a group of important RNA viruses, have a significant impact on DCs that cannot be ignored. Influenza viruses belong to the Orthomyxoviridae family, with genetic material composed of single-stranded negative-sense RNA, and they are known for their high variability. Influenza viruses are classified into multiple subtypes based on antigenic differences in the surface proteins hemagglutinin (HA) and neuraminidase (NA). The interaction between the influenza virus and host PRRs constitutes the core mechanism initiating antiviral immune responses, as the viral PAMPs are recognized by multiple PRRs. During infection with IAV, DCs activate antiviral responses by recognizing viral single-stranded RNA and dsRNA intermediates through their PRRs, such as TLR7 and 3, respectively [47,59]. Concurrently, TLR4 facilitates the upregulation of co-stimulatory molecules CD40 and CD86, driving DC maturation. TLR4 signaling further induces the secretion of pro-inflammatory cytokines such as IL-6 and TNF-α while enabling the cross-presentation of antigens to activate CD8^+^ T cells [122]. SIGN-R1, functioning as a CLR of PRRs, specifically recognizes glycosylated structures on the influenza viral surface, mediating the capture of inactivated viral particles by nasal mucosal DCs and thereby enhancing antigen presentation [102]. The activation of these receptors promotes the production of IFN-I, which is a key factor in controlling virus replication and spread [184]. In the early stages of influenza virus infection, DCs, through the expression of TLR7, sense viral RNA and activate transcription factor IRF7 through MyD88-dependent pathways, thereby inducing IFN-I production [185]. DCs can also recognize viral RNA through the endosomal pathway associated with the endoplasmic reticulum, such as TLR3, further promoting IFN-I production [186]. Influenza virus infection can induce DCs to produce inflammatory factors such as TNF-α and IL-6, which play a role in regulating the inflammatory response and attracting other immune cells to the infection site [187]. Influenza virus may also interfere with DCs’ antigen-presenting functions, reducing their ability to express MHC-II and co-stimulatory molecules, thereby weakening DCs’ ability to activate T cells [188].

H1N1, H5N1, and H9N2 are among the subtypes with significant public health importance. The H1N1 influenza virus has caused multiple influenza pandemics in history, with a wide host range including humans and various animals [189]. The genetic characteristics of this virus include gene plasticity and a strong ability to reassort, allowing it to adapt to different host environments and evade the host’s immune defenses. The H5N1 influenza virus, commonly known as highly pathogenic avian influenza (HPAI), primarily infects birds, especially poultry, but can also cross species barriers to infect humans and other mammals [190]. The genome of the H5N1 virus contains multiple gene markers associated with viral pathogenicity and transmissibility. Its high mutation and reassortment rates increase its threat to public health [191]. The H9N2 influenza virus is a low-pathogenic avian influenza virus (LPAIV) that has drawn attention due to its infections in humans and other mammals [192]. H9N2 viruses exhibit high genetic compatibility with the internal genes of H5N1 and H7N9 viruses, which may be related to their ability to transmit and adapt between different hosts. The impact of these influenza virus subtypes on DCs is primarily reflected in their ability to interfere with the maturation and function of DCs through various mechanisms, thereby affecting the host’s immune response. For example, viruses can evade immune surveillance by inhibiting the antigen-presenting ability of DCs, reducing the production of IFNs, or promoting the differentiation of regulatory T cells [193]. DCs, as key members of the immune system, play a crucial role in the immune response to RNA viruses in zoonoses. In particular, the H1N1, H5N1, and H9N2 subtypes of influenza viruses activate the immune response of DCs by specifically recognizing and infecting host cells. The H1N1 influenza virus primarily binds to sialic acid receptors on the host cell surface through its surface glycoprotein HA protein, triggering endocytosis of the virus. Subsequently, within the endosome with low pH, HA undergoes a conformational change that promotes the fusion of the viral envelope with the endosomal membrane, releasing the viral nucleic acid into the cytoplasm [189]. The H5N1 virus, due to its high pathogenicity, can infect various cell types through a similar mechanism, but its infectivity and pathogenicity are closely related to the type and density of receptors on the host cell surface [190]. Although H9N2 viruses are generally considered to be of low pathogenicity, their infection mechanism is similar to that of H1N1 and H5N1, relying on HA-mediated receptor recognition and membrane fusion [192]. After the virus enters the host cell, DCs recognize viral components through PRRs, activate downstream signaling pathways, and promote the production of IFN-I and other cytokines, thereby initiating the antiviral immune response. Viral nucleoprotein (NP) and nonstructural protein 1 (NS1), among other components, may also affect the maturation and antigen-presenting function of DCs, thereby regulating the intensity and direction of the immune response. NS1–host interactions are species-restricted: human/murine NS1 binds RIG-I’s CARD domain, whereas avian NS1 suppresses MDA5 signaling in chickens, demonstrating pathogen adaptation to host-specific pathways [194,195,196]. Influenza A virus subtypes H1N1, H5N1, and H9N2 enter host cells through HA-mediated receptor recognition and endosomal fusion mechanisms. DCs activate the immune response by recognizing viral components, while specific proteins of the virus may impact the function of DCs. These mechanisms collectively determine the host’s immune response to influenza virus infection [197].

The H1N1 virus can infect human DCs and trigger viral endocytosis by binding its surface protein, the HA protein, to specific receptors on DCs [198]. This interaction not only facilitates viral spread but may also regulate the host’s immune response by affecting DC maturation and antigen-presenting function. The interaction between the highly pathogenic avian influenza virus H5N1 and DCs is more complex. H5N1 can infect DCs by its HA protein binding to the CLR receptor DC-SIGN, thereby promoting viral infection [199]. The N-glycosylation sites of the H5N1 virus, particularly those at N27 and N39, play a crucial role in its interaction with DC-SIGN, significantly weakening viral binding and infectivity. This interaction not only affects viral transmission within the host but may also have a significant impact on viral pathogenicity and the host’s immune response. The H9N2 subtype, an LPAIV prevalent in birds, also interacts with DCs. H9N2 can infect chicken DCs and induce changes in miRNA expression, which plays a key role in regulating the host’s immune response and viral replication [200]. For example, miRNAs such as miR-155 and miR-130b-3p are considered to have antiviral activity, affecting immune response and regulating viral replication by targeting specific genes in host cells. H1N1, H5N1, and H9N2 subtypes use their surface HA protein to bind to sialic acid receptors on host cells, enabling virus attachment and endocytosis. H1N1 typically causes seasonal influenza, while H5N1 and H9N2 are of concern due to their threat to humans and poultry. H5N1 binds to receptors on the DC surface through its highly conserved region in the HA protein, which not only promotes viral endocytosis but may also affect DC function by inhibiting maturation and migration. H5N1 can induce higher levels of IFN-I but also upregulates PD-1 expression, which may lead to T-cell function suppression and delayed virus clearance [201]. H5N1 can also induce high levels of the anti-inflammatory cytokine IL-10, which may further inhibit DC function and T-cell response. In contrast, while H1N1 can interact with DCs through its HA protein, the immune response it elicits is relatively weak and typically does not cause severe immunopathology [202]. Less is known about the impact of H9N2, with its lower pathogenicity, on DCs and immune response mechanisms; however, its infection may still regulate the host’s immune response by affecting DC activation status and antigen-presenting capability [203]. The interaction between influenza viruses and DCs also involves viral replication and spread. After entering DCs through endocytosis, the virus’s replication process may be influenced by the DC’s internal environment [204]. For instance, the replication of the H5N1 virus within DCs may result in higher viral loads, which in turn may exacerbate the disease’s severity by promoting further viral spread. The viral regulation of DC function may affect their ability to migrate to lymph nodes, thus impacting T-cell activation and differentiation and subsequently affecting the overall quality and efficiency of the adaptive immune response. Influenza viruses, by interacting with receptors on the surface of DCs, not only affect viral replication and spread but may also influence the host’s immune response by modulating the function of DCs.

The H5N1 influenza virus exhibits high pathogenicity and significant pandemic potential, which differs from that of seasonal H1N1 strains. During H5N1 infection, DCs recognize the virus primarily through TLRs and RLRs, triggering robust downstream signaling (e.g., NF-κB and IRF3 pathways) [205]. This response drives excessive DC maturation and hyperactivation, leading to elevated expression of antigen-presenting molecules (e.g., MHC-II, CD80/86) and contributing to dysregulated cytokine production (“cytokine storm”), a hallmark of H5N1 pathogenesis [123,206]. The H5N1 virus binds to specific receptors on DCs through its HA and NA proteins, which may affect DC activation and maturation processes [207]. Chen et al. demonstrated that amino acids 80 to 84 of the NS1 protein enhanced the expression of phenotypic markers (CD80, CD86, CD40, and MHCII), the activation marker CD69, and inflammatory cytokines (IL-6, TNF-α, and IL-10) while antagonizing IFN-α in DCs. H5N1 virus with no deletion of amino acids 80 to 84 of the NS1 protein induced DCs to quickly migrate into nearby cervical lymph nodes by highly upregulating CCR7, and CD86 showed high expression on the migrated DCs. These findings suggest that amino acids 80 to 84 of the NS1 protein strongly induce the innate immune response, which is conducive to activating a broad immune response, resulting in a strong cytokine storm and enhancing the pathogenicity of H5N1 subtype AIVs in mammals [105].

The H9N2 subtype of influenza virus, as an LPAIV, is widely spread among chicken flocks and poses a potential threat to humans. The H9N2 virus can induce DCs to produce inflammatory factors, promoting viral infection and spread through interactions with receptors such as DC-SIGN [208]. The impact of the H9N2 virus on DCs may be related to its transmission and evolution within the host, especially in the process of gene reassortment, where it provides internal genes to other novel influenza viruses. During influenza virus infection, the activation and maturation status of DCs is crucial for the subsequent T-cell response. Mature DCs can migrate to lymph nodes and present viral antigens to T cells through MHC molecules, thereby activating specific cellular immune responses. Influenza viruses employ various strategies to interfere with DC function, potentially affecting the effective presentation of antigens and the activation of T cells, thereby influencing the host’s immune protection. The impact of influenza viruses on DCs is multifaceted, encompassing activation, maturation, antigen presentation, and interactions with host immune cells [209].

Qin et al. demonstrated that the PA-X protein inhibited H9N2 subtype AIVs from infecting DCs but not MDCK cells. Meanwhile, the PA-X protein suppressed the phenotypic expression (CD80, CD86, CD40, and MHCII), early activation marker (CD69), and pro-inflammatory cytokines (IL-6 and TNF-α) but increased anti-inflammatory cytokine (IL-10) levels in DCs. After intranasal viral infection in mice, we found that the PA-X protein of H9N2 subtype AIVs reduced the recruitment of CD11b^+^ and CD103^+^ subtype mucosal DCs to the nasal submucosa by inhibiting CCL20 expression. Moreover, the PA-X protein abolished the migratory ability of CD11b^+^ and CD103^+^ DCs into draining cervical lymph nodes by down-regulating CCR7 expression [124]. In chicken bone marrow-derived DCs (chBM-DCs), the PA-X protein also suppressed the innate immune response, which helped H9N2 subtype AIVs escape the innate immunity of chBM-DCs [210]. We also demonstrated that the PA-X protein played a critical role in evading the immune response of nasal mucosal DCs, thereby increasing the virulence of H1N1 viruses. Following intranasal infection in mice, CCL20, a chemokine that monitors the recruitment of submucosal DCs, was downregulated by PA-X, resulting in an inhibition of the recruitment of CD11b^+^ DCs to the submucosa. It also attenuated the migration of CCR7^+^ DCs to cervical lymph nodes and inhibited DC maturation, characterized by low MHC II and CD40 expression [125].

H5N1 virus can inhibit the maturation of certain DC subsets, thereby reducing the expression of co-stimulatory molecules and the production of cytokines, which may lead to insufficient T-cell activation and weaken the host’s adaptive immune response [207]. The H5N1 virus can also activate specific DC subsets through certain mechanisms, triggering a strong immune response that may be related to viral pathogenicity and the host’s immunopathological processes. The impact of the H9N2 virus, as an LPAIV, on DCs has been less studied. However, existing studies have shown that the H9N2 virus can induce DCs to produce a certain level of cytokines, such as IL-6 and IL-12, which help promote the differentiation and activation of T cells [208]. The activation of DCs by the H9N2 virus may also involve the recognition of PRRs, such as TLRs and RLRs, which contribute to the promotion of the antiviral response.

Different subtypes of influenza viruses affect the immune regulatory function of DCs through various mechanisms, including the production of cytokines, the expression of co-stimulatory molecules, and the activation of T cells. Infections with H1N1 and H5N1 viruses significantly promote the maturation and activation of DCs, enhancing their expression of co-stimulatory molecules such as CD80 and CD86, thereby effectively activating T cells. These viral infections can also induce DCs to produce cytokines such as IFN-γ and TNF-α, which are crucial for activating and regulating the T-cell immune response. The H9N2 subtype of the influenza virus affects DC function through its NA. The NA of the H9N2 virus can unmask CD83 on the cell surface and enhance cytokine production [211]. CD83 is a highly expressed transmembrane glycoprotein on mature DCs that plays a crucial role in the activation and proliferation of T cells. CD83 is also a sialylated glycoprotein. NA treatment resulted in the sialic acid modification of the CD83 protein and enhanced LPS-stimulated NF-κB activation in RAW264.7 cells. Anti-CD83 treatment alleviated influenza virus-induced lung injury in mice [211]. The homotypic interaction of CD83 regulates DC activation via mitogen-activated protein kinase (MAPK) by inhibiting p38α phosphorylation [212]. The anti-CD83 antibody would presumably block this homotypic interaction. NA treatment may induce this homotypic interaction of CD83. In the case of H5N1 and H9N2 virus infections, the activation and maturation of DCs may be affected to varying degrees, which may influence their role in virus clearance and disease progression. For example, the H5N1 virus may weaken the host’s immune response by affecting certain functions of DCs, while the H9N2 virus may enhance the immune response through the immunomodulatory effects of NA [192]. Influenza virus subtypes H1N1, H5N1, and H9N2 have complex effects on DC-mediated immune responses, which may determine the efficiency of virus clearance and the course of disease development.

## 6. Conclusions

As APCs, DCs are capable of effectively recognizing viral components and presenting antigens to T cells through MHC molecules, activating T-cell-mediated immune responses [213]. The maturation and migration capabilities of DCs are essential for delivering antigens from DNA and RNA viruses to T cells and initiating adaptive immune responses [214].

The prevention and control of zoonoses face many challenges, including the rapid mutation of pathogens, the complexity of the host immune response, and the diversity of pathogen transmission [215]. DCs play a key role in this process, not only because of their core function in initiating immune responses but also because of their role in regulating immune reactions [216]. The activation and maturation status of DCs directly affects the activation and differentiation of T cells, which are crucial for controlling and clearing zoonotic pathogens [217]. The regulatory function of DCs helps maintain immune balance and prevent pathological damage caused by excessive immune responses [218]. DCs have significant potential applications in the prevention, treatment, and development of vaccines for zoonoses.

Future research should focus on elucidating the specific mechanisms of DCs in the immune response to DNA and RNA viruses, including how they recognize PAMPs of different types of viruses and how they activate immune cells through various signaling pathways. Studying the role of DCs in various zoonotic models will help us understand their immune regulatory functions in both natural and accidental hosts. In terms of clinical applications, the development of DC-based vaccine strategies requires further optimization, including enhancing the antigen-presenting capacity of DCs, improving the immune response, and developing personalized DC vaccines targeting specific pathogens [121]. Exploring the interactions between DCs and other immune cells, such as B cells and T cells, will aid in the development of comprehensive immunotherapy strategies and enhance the prevention and control of zoonoses.

DCs face inherent physiological constraints that affect their ability to control zoonotic diseases. Constituting a mere 0.1–0.5% of human peripheral blood leukocytes [8], DC scarcity creates immunosurveillance gaps at mucosal portals of viral entry—such as respiratory and intestinal tracts—enabling pathogens such as SARS-CoV-2 to exploit these vulnerabilities through ORF8-mediated MHC-I suppression [219]. At the same time, the influenza A virus hijacks DC-SIGN for systemic dissemination [220]. To counter these limitations, three synergistic strategies are emerging: amplifying endogenous DC pools via Flt3L administration [221], engineered recruitment using chemokine-functionalized nanoparticles [222], and directing immune bias toward protective responses through TLR3/RIG-I agonists combined with IL-10 blockade to prevent regulatory DC differentiation [223]. Integrating these approaches with species-specific targeting could transform DC biology into actionable tools for pandemic preparedness and response.

## Data Availability

No new data were created or analyzed in this study. Data sharing does not apply to this study.
